# Supplementary material for: Efficient Photodynamic Therapy of Prostate Cancer Cells through an Improved Targeting of the Cation-Independent Mannose 6-Phosphate Receptor
Source: Int J Mol Sci. 2019 Jun 8;20(11):2809. doi: 10.3390/ijms20112809 (PMC6600508; doi:10.3390/ijms20112809)
Supplement: Supplementary file 1 [file ijms-20-02809-s001.pdf]

Electronic Supplementary Information for

Efficient photodynamic therapy of prostate cancer cells through an improved targeting of the cation-independent mannose 6-phosphate receptor.

Elise Bouffard[1], Chiara Mauriello Jimenez[2], Khaled El Cheikh[3], Marie Maynadier[3], Ilaria Basile[3], Laurence Raehm[2], Christophe Nguyen[1], Magali Gary-Bobo*[1], Marcel Garcia[1], Jean-Olivier Durand*[2] and Alain Morère*[1]

[1] *IBMM, Univ Montpellier, CNRS, ENSCM, Montpellier, France*

[2] *ICGM, Univ Montpellier, CNRS, ENSCM, Montpellier, France*

[3] *NanoMedSyn, Avenue Charles Flahault, 34093 Montpellier cedex 05 (France)*

Table of contents

General remarks 3

Synthesis and characterization of saccharides 3

Synthesis of nanoparticles 16

Characterization of the nanoparticles (Figure S1, Figure S2, Figure S3) 18

General remarks

Reactions were conducted under nitrogen atmosphere. Reagents were purchased from Sigma. All solvents were distilled prior to use, methylene chloride was distilled over P_2O_5, methanol was distilled over sodium and THF was distilled over sodium-benzophenone. Optical rotations were measured with a Perkin Elmer 241 polarimeter (sodium D line: 589 nm). NMR spectra were recorded with a Brüker DRX 400 (400 MHz for ^1H, 100 for ^{13}C) and Brüker DRX 600 (600 MHz for ^1H, 150 for ^{13}C) instrument with Me_4Si (δ=0 ppm) as internal standard. Flash column chromatography was performed on silica gel 60 (230–400 mesh, Merck). Purifications on RP-Gel were carried out using reversed phase silica gel 100 (C18, Fluka) columns. ESI MS spectra were measured on a Waters Micromass apparatus.

Synthesis and characterization of saccharides

2-bromoethyl *α*-D-mannopyranoside (1)

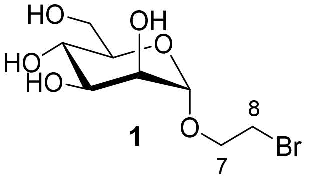

D-mannose (15 g, 83.3 mmol) was dissolved in 2-bromoethanol (45 mL, 635 mmol) with Amberlite IR120 resin (15 g). The slurry is warmed up to 80°C and stirred for 3h30. After cooling to RT, the suspension is filtered. The filtrate is concentrated and purify on silica gel (CH_2Cl_2/MeOH 0 to 15%) to give compound **1** (54%).

^{1}H NMR (600 MHz, D_2O, 298 K): δ 3.71-3.63 (m, 3H, H-4, H-8), 3.80-3.75 (m, 2H, H-5, H-7), 3.85 (dd, $^3J_{H3-H4}$ = 9.6 Hz, $^3J_{H3-H2}$ = 3.4 Hz, 1H, H-3), 3.95-3.89 (m, 2H, H-6, H-7′), 4.01 (dd, $^3J_{H2-H3}$ = 3.4 Hz, $^3J_{H1-H2}$ = 1.7 Hz, 1H, H-2), 4.08-4.04 (m, 1H, H-6′), 4.96 (d, $^3J_{H1-H2}$ = 1.7 Hz, 1H, H) ppm

^{13}C NMR (100 MHz, MeOD, 298K) δ 31.5 (1C, C-8), 62.9 (1C, C-6), 68.5 (1C), 68.9 (1C, C-7), 72.0 (1C, C-2), 72.5 (1C), 75.0 (1C), 101.8 (1C, C-1) ppm

MS, ESI$^-$ *m/z*: 331 [**M**-H+HCOOH]$^-$

2-azidoethyl 2,3,4-tri-*O*-trimethylsilyl-*α*-D-mannopyranoside (2)

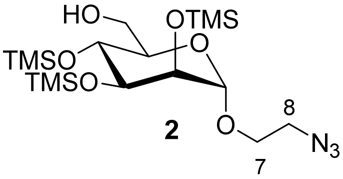

2-bromoethyl *α*-D-mannopyranoside **1** (2 g, 6.97 mmol) was dissolved in DMF (17 mL). Sodium azide (543 mg, 8.36 mmol) was added. After 20 h of stirring at RT, the solution was concentrated to dryness.

The residue (1.74 g, 6.97 mmol) was dissolved in CH_2Cl_2 (20 mL) and triethylamine (28.2 mL, 209.1 mmol). The solution was cooled to 0°C and TMSCl (5.1 mL, 55.76 mmol) was added dropwise. After 20h of stirring at RT, the solution was concentrated. The crude was taken in cyclohexane and filtered over celite. The filtrate was concentrated.

The persilylated compound (3.48 g, 6.47 mmol) was dissolved in methanol (3.5 mL). A solution of potassium carbonate (9 mg, 0.067 mmol) in methanol (71.5 mL) was added at 0°C. The solution was stirred at 0°C for 50 min and then diluted with CH_2Cl_2 (180mL) and washed with brine. The aqueous layer was extracted with CH_2Cl_2. The combined organic layers were dried over sodium sulfate and concentrated. The crude was purified on silica gel (cyclohexane/diethylether 0 to 30%) to give the compound **2** (38% over 3 steps).

^{1}H NMR (400 MHz, $CDCl_3$, 298K) δ 0.14 (s, 9H, CH_3-Si), 0.15 (s, 9H, CH_3-Si), 0.15 (s, 9H, CH_3-Si), 1.97 (dd, 1H, OH, $^3J_{OH\text{-}H6}$ = 5.9Hz, $^3J_{OH\text{-}H6'}$ = 6.7Hz, O<u>H</u>), 3.33 (ddd, 1H, $^3J_{H8'\text{-}H7'}$ = 3.6Hz, $^3J_{H8'\text{-}H7}$ = 5.8Hz, $^2J_{H8'\text{-}H8}$ = 13.3Hz, H-8′), 3.38 (ddd, 1H, $^3J_{H8\text{-}H7}$ = 3.4Hz, $^3J_{H8\text{-}H7'}$ = 6.7Hz, $^2J_{H8\text{-}H8'}$ = 13.3Hz, H-8), 3.57 (ddd, 1H, $^3J_{H5\text{-}H6'}$ = 2.8Hz, $^3J_{H5\text{-}H6}$ = 5.3Hz, $^3J_{H5\text{-}H4}$ = 8.9Hz, H-5), 3.59 (ddd, 1H, $^3J_{H7'\text{-}H8}$ = 3.6Hz, $^3J_{H7'\text{-}H8}$ = 6.7Hz, $^2J_{H7'\text{-}H7}$ = 10.5Hz, H-7′), 3.70 (ddd, 1H, $^3J_{H6'\text{-}H5}$ = 2.8Hz, $^3J_{H6'\text{-}OH}$ = 5.9Hz, $^2J_{H6'\text{-}H6}$ = 11.9Hz, H-6′), 3.77 (ddd, 1H, $^3J_{H6\text{-}H5}$ = 5.3Hz, $^3J_{H6\text{-}OH}$ = 6.7Hz, $^2J_{H6\text{-}H6'}$ = 11.9Hz, H-6), 3.79 (dd, 1H, $^3J_{H3\text{-}H2}$ = 2.6Hz, $^3J_{H3\text{-}H4}$ = 8.9Hz, H-3), 3.83 (dd, 1H, $^3J_{H2\text{-}H1}$ = 1.8Hz, $^3J_{H2\text{-}H3}$ = 2.6Hz, H-2), 3.87 (t, 1H, $^3J_{H4\text{-}H3}$ = $^3J_{H4\text{-}H5}$ = 8.9Hz, H-4), 3.89 (ddd, 1H, $^3J_{H7\text{-}H8}$ = 3.4Hz, $^3J_{H7\text{-}H8'}$ = 5.8Hz, $^2J_{H7\text{-}H7'}$ = 10.5Hz, H-7), 4.65 (d, 1H, $^3J_{H1\text{-}H2}$ = 1.8Hz, H-1) ppm
^{13}C NMR (100 MHz, $CDCl_3$, 298K) δ 0.4 (3C, <u>C</u>H_3-Si), 0.8 (3C, <u>C</u>H_3-Si), 0.8 (3C, <u>C</u>H_3-Si), 50.7 (1C, C-8), 62.4 (1C, C-6), 66.9 (1C, C-7), 68.1 (1C, C-4), 72.4 (1C, C-3), 73.6 (1C, C-2), 74.5 (1C, C-5), 101.4 (1C, C-1) ppm

MS, ESI$^+$ *m/z* : 488.2 [**M**+Na]$^+$

2-azidoethyl (6E)-6,7-dideoxy-7-ethoxycarbonyl-2,3,4-tri-*O*-trimethylsilyl-α-D-*manno*-hept-6-enopyranoside (3)

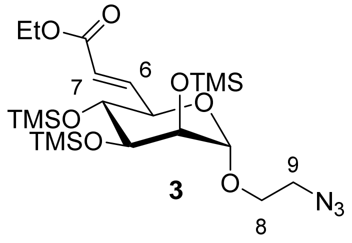

Compound **2** (200 mg, 0.43 mmol) was dissolved in CH_2Cl_2 (9 mL). Dess-Martin periodinane solution 0.3M in CH_2Cl_2 (2.17 mL, 0.65 mmol) was added. The solution was stirred at RT for 1 h and then diluted with Et_2O (9 mL). A solution of $NaHCO_3$ (9 mL) and sodium thiosulfate (1.56 g) were added. After 30 min of stirring at RT, the solution was diluted with Et_2O (40 mL). The aqueous layer was extracted with Et_2O (4 x 20 mL). The combined organic layers were washed with water (40 mL), dried ($MgSO_4$) and concentrated to afford the aldehyde directly used for next step.

NaH 60% dispersion in oil (84 mg, 2.5 mmol) was dissolved in THF (15 mL). Triethylphosphonoacetate (397 μL, 2 mmol) was added dropwise. After 45 min of stirring at RT, the solution was added to the aldehyde (232 mg, 0.5 mmol) dissolved in THF (3.5

mL). The solution was stirred at RT for 1 h, diluted with CH_2Cl_2 (80 mL) and washed with brine (2x 25 mL). The aqueous layer was extracted with CH_2Cl_2 (60 mL). The combined organic layers were dried ($MgSO_4$) and concentrated. The residue was purified by flash chromatography (cyclohexane/diethylether 0 to 30%) to give the compound **3** in 72% yield over 2 steps.

^{1}H NMR (400 MHz, CDCl$_3$, 298K) δ 0.11 (s, 9H, CH_3-Si), 0.14 (s, 9H, CH_3-Si), 0.15 (s, 9H, CH_3-Si), 1.28 (t, 3H, C<u>H</u>$_3$-CH$_2$-O, $^3J_{H\text{-}H}$ = 7.1Hz), 3.32-3.36 (m, 2H, H-9), 3.59 (ddd, 1H, $^3J_{H8\text{-}H9'}$ = 4.3Hz, $^3J_{H8\text{-}H9}$ = 5.9Hz, $^2J_{H8\text{-}H8'}$ = 10.6Hz, H-8), 3.71 (t, $^3J_{H4\text{-}H3}$ = $^3J_{H4\text{-}H5}$ = 9.2Hz, 1H, H-4), 3.79 (dd, 1H, $^3J_{H3\text{-}H2}$ = 2.6Hz, $^3J_{H3\text{-}H4}$ = 9.2Hz, H-2), 3.84 (dd, 1H, $^3J_{H2\text{-}H1}$ = 1.9Hz, $^3J_{H2\text{-}H3}$ = 2.6Hz, H-2), 3.84 (ddd, $^3J_{H8'\text{-}H9}$ = 4.3Hz, $^3J_{H8'\text{-}H9'}$ = 5.3Hz, $^2J_{H8'\text{-}H8}$ = 10.6Hz, H-8'), 4.13 (ddd, $^3J_{H5\text{-}H7}$ = 1.7Hz, $^3J_{H5\text{-}H6}$ = 5.0Hz, $^3J_{H5\text{-}H4}$ = 9.2Hz, 1H, H-5), 4.16-4.25 (m, 2H, CH$_3$-C<u>H</u>$_2$-O), 4.67 (d, $^3J_{H1\text{-}H2}$ = 1.9Hz, 1H, H-1), 6.14 (dd, $^4J_{H7\text{-}H5}$ = 1.7Hz, $^3J_{H7\text{-}H6}$ = 15.7 Hz, 1H, H-7), 7.04 (dd, $^3J_{H6\text{-}H5}$ = 5.0Hz, $^3J_{H6\text{-}H7}$ = 15.7Hz, 1H, H-6) ppm
^{13}C NMR (100 MHz, CDCl$_3$, 298K) δ 0.4 (s, 3C, <u>C</u>H$_3$-Si), 0.8 (s, 3C, <u>C</u>H$_3$-Si) , 0.9 (s, 3C, <u>C</u>H$_3$-Si), 14.4 (s, 1C, <u>C</u>H$_3$-CH$_2$-O), 50.7 (s, 1C, C-9), 60.4 (s, 1C, CH$_3$-<u>C</u>H$_2$-O), 66.9 (s, 1C, C-8), 71.6 (s, 1C, C-4), 72.6 (s, 1C, C-3), 73.1 (s, 1C, C-5), 73.4 (s, 1C, C-2), 101.4 (s, 1C, C-1), 122.0 (s, 1C, C-7), 145.2 (s, 1C, C-6), 166.4 (s, 1C, C=O) ppm

MS, ESI$^+$ *m/z* : 556.2 [**M**+Na]$^+$

2-aminoethyl 6,7-dideoxy-7-hydroxycarbonyl-α-D-*manno*-heptopyranoside (4)

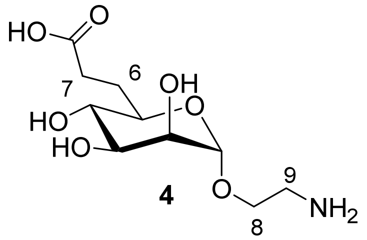

To a solution of **3** (200 mg, 0.375 mmol) in THF (5.5 mL), were added 2.25 mL (1.12 mmol) of an aqueous solution of HCl 0.5N. After 15 min of stirring at RT, the solution was neutralized with a saturated solution of $NaHCO_3$ and concentrated. Salts were precipitated in cold methanol and the filtrate was concentrated. The residue was dissolved in NaOH 0.1N (3.75 mL, 0.375 mmol). The solution was stirred at RT for 30 min and then neutralized with HCl 0.5N and concentrated. The residue was purified on C18 silica gel (water 100%) to give the title compound in 99% yield.

The deprotected compound (57 mg, 0.2 mmol) was dissolved in a mixture ethanol/water (2:1) (4 mL). 10% palladium on charcoal (6 mg) was added and the suspension was stirred at RT under hydrogen flux for 1 h 30. The suspension was filtered over celite and the

filtrate was concentrated to give the compound **4**, used for next step without further purification.

^{1}H NMR (400 MHz, D_2O, 298K) δ 1.75 (dtd, 1H, $^3J_{H6'-H7}$ = 5.6Hz, $^3J_{H6'-H7'}$ = $^3J_{H6'-H5}$ = 8.0Hz, $^2J_{H6'-H6}$ = 14.3Hz, H-6′), 2.21 (dddd, 1H, $^3J_{H6-H5}$ = 1.9Hz, $^3J_{H6-H7'}$ = 8.0Hz, $^3J_{H6-H7}$ = 8.9Hz, $^2J_{H6-H6'}$ = 14.3Hz, H-6), 2.35 (dt, 1H, $^3J_{H7'-H6'}$ = $^3J_{H7'-H6}$ = 8.0Hz, $^2J_{H7'-H7}$ = 15.2Hz, H-7′), 2.46 (ddd, 1H, $^3J_{H7-H6'}$ = 5.6Hz, $^3J_{H7-H6}$ = 8.9Hz, $^2J_{H7-H7'}$ = 15.2Hz, H-7), 3.05-3.18 (m, 2H, H-9), 3.48-3.61 (m, 2H, H-4, H-5), 3.62-3.70 (m, 1H, H-8), 3.90-3.95 (m, 1H, H-8′), 3.85 (dd, 1H, $^3J_{H3-H2}$ = 3.5Hz, $^3J_{H3-H4}$ = 9.5Hz, H-3), 4.03 (dd, $^3J_{H2-H1}$ = 1.6Hz, $^3J_{H2-H3}$ = 3.5Hz, 1H, H-2), 4.89 (d, $^3J_{H1-H2}$ = 1.6Hz, 1H, H-1) ppm

^{13}C NMR (100 MHz, D_2O, 298K) δ 27.7 (s, 1C, C-6), 33.5 (s, 1C, C-7), 40.0 (s, 1C, C-9), 68.0 (s, 1C, C-8), 70.3 (s, 1C, C-2), 70.7 (s, 2C, C-3 + C-4), 71.9 (s, 1C, C-5), 99.9 (s, 1C, C-1), 182.8 (s, 1C, C=O) ppm

MS, ESI$^+$ *m/z* : 266.0 [**M**+H]$^+$

(4-ethoxy-2,3-dioxocyclobut-1-enyl)-2-aminoethyl 6,7-dideoxy-7-hydroxycarbonyl-*α*-D-*manno*-heptopyranoside (5)

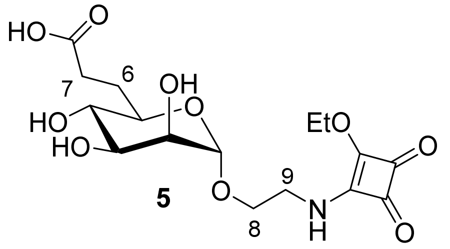

To diethylsquarate (48 μL, 0.42 mmol) diluted in a mixture ethanol/water (2:1) was added dropwise a solution of compound **4** (100 mg, 0.38 mmol) in ethanol/water (2:1) (2.5 mL). After 40 min of stirring at RT, the solution was concentrated and the residue was purified on C18 (water 100%) to give the title compound **5** as a white solid in 34% yield.

$[\alpha]_D^{20}$ = 0° (c=5.3.10^{-3}M/H_2O)

^{1}H NMR (400 MHz, D_2O, 353K) δ 1.94 (t, 3H, $^3J_{H-H}$ = 7.0Hz, $C\underline{H}_3$-CH_2-O), 2.21 (dddd, 1H, $^3J_{H6-H5}$ = 2.5Hz, $^3J_{H6-H7}$ = 6.8Hz, $^3J_{H6-H7'}$ = 9.4Hz, $^2J_{H6-H6'}$ = 14.2Hz, H-6), 2.58 (dtd, 1H, $^3J_{H6'-H7'}$ = 5.8Hz, $^3J_{H6'-H7}$ = $^3J_{H6'-H5}$ = 9.0Hz, $^2J_{H6'-H6}$ = 14.2Hz, H-6′), 2.73 (ddd, 1H, $^3J_{H7-H6}$ = 6.8Hz, $^3J_{H7-H6'}$= 9.0Hz, $^2J_{H7-H7'}$ = 15.1Hz, H-7), 2.87 (ddd, 1H, $^3J_{H7'-H6'}$ = 5.8Hz, $^3J_{H7'-H6}$= 9.4Hz, $^2J_{H7'-H7}$ = 15.1Hz, H-7′), 3.95 (dt, 1H, $^3J_{H5-H6}$ = 2.5Hz, $^3J_{H5-H4}$ = $^3J_{H5-H6'}$ = 9.5Hz, H-5), 4.00 (t, 1H, $^3J_{H4-H3}$ = $^3J_{H4-H5}$ = 9.5Hz, H-4), 4.14 (dd, 1H, $^3J_{H3-H2}$ = 3.2Hz, $^3J_{H3-H4}$ = 9.5Hz, H-4), 4.16-4.31 (m, 4H, H-8, H-8′, H-9, H-9′), 4.36 (dd, 1H, $^3J_{H2-H1}$ = 1.5Hz, $^3J_{H2-H3}$ = 3.2Hz, H-2), 5.30 (d, 1H, $^3J_{H1-H2}$ = 1.5Hz, H-1), 5.24 (q, 2H, $^3J_{H-H}$ = 7.0Hz, CH_3-$C\underline{H}_2$-O) ppm

^{13}C NMR (100 MHz, D_2O, 353K) δ 15.6 (1C, $\underline{C}H_3$-CH_2-O), 27.7 (s, 1C, C-6), 32.9 (1C, C-7), 44.6 (1C, C-10), 66.9 (1C, C-9), 69.90, 69.93 (2s, 1C), 71.4 (1C, CH_3-$\underline{C}H_2$-O), 70.33, 70.45 (2s,

1C), 70.8 (1C, C-2), 71.0 (1C, C-4), 71.4 (1C, C-3), 72.6 (1C, C-5), 100.3 (1C, C-1), 174.8 (1C, C=C-NH), 178.2 (1C, C=C-OEt), 180.0 (1C, HO-C=O), 184.2-184.6 (1C, C-13), 189.8-189.0 (1C, C-12) ppm

HRMS : calculated mass: 388.1244, found: 388.1249

1,2-*O*-(1-(2-bromoethoxy)ethylidene)-3,4,6-tri-*O*-acetyl-α-D-mannopyranose (6)

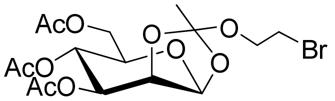

6

Pentaacetylated α-D-mannose (10 g, 25.6 mmol) was dissolved in a solution of bromhydric acid 33% in acetic acid (20 mL). The solution was stirred at RT for one hour and then cooled to 0°C and diluted with CH_2Cl_2 (30 mL). The solution was neutralized with a saturated aqueous solution of $NaHCO_3$. The aqueous layer was extracted with CH_2Cl_2 (5x100 mL). The combined organic layers were dried ($MgSO_4$) and concentrated.

The residue (11.08 g, 26.9 mmol) was dissolved in CH_2Cl_2 (9.5 mL) and 2,6-lutidine (9.4 mL, 80.7 mmol). Then, 2-bromoethanol (4.8 mL, 67.25 mmol) was added. The solution was stirred at 40°C for 4 h. The solution was cooled down to RT and Et_2O (12 mL) was added. The precipitate was filtered and the filtrate was diluted with CH_2Cl_2 (30 mL) and washed successively with water (30 mL), a saturated aqueous solution of $NaHCO_3$ (30 mL) and brine (30 mL). The combined aqueous layers were extracted with CH_2Cl_2 (50 mL). The combined organic layers were dried ($MgSO_4$) and concentrated. The residue was dissolved in CH_2Cl_2 and ethanol (25 mL) was added. The solution was cooled to -30°C to give the compound **6** as white crystals (4.93 g, 56%).

^{1}H NMR (400 MHz, CDCl$_3$, 298K) δ 1.76 (s, 3H, CH_3), 2.06 (s, 3H, CH_3CO), 2.08 (s, 3H, CH_3CO), 2.13 (s, 3H, CH_3CO), 3.43 (t, $^3J_{H-H}$ = 6.3Hz, 2H, CH_2Br), 3.70 (ddd, 1H, $^3J_{H5-H4}$ = 9.4Hz, $^3J_{H5-H6}$ = 5.0Hz, $^3J_{H5-H6'}$ = 2.7Hz, H-5), 3.81 (td, 2H, $^3J_{H-H}$ = 6.3Hz, $^3J_{H-H}$ = 1.2Hz, 2H, CH_2O), 4.15 (dd, $^2J_{H6'-H6}$ = 12.2Hz, $^3J_{H6'-H5}$ = 2.7Hz, 1H, H-6'), 4.24 (dd, $^2J_{H6-H6'}$ = 12.2Hz, $^3J_{H6-H5}$ = 5.1Hz, 1H, H-6), 4.65 (dd, $^3J_{H2-H3}$ = 3.9Hz, $^3J_{H2-H1}$ = 2.7Hz, 1H, H-2), 5.17 (dd, $^3J_{H3-H4}$ = 9.9Hz, $^3J_{H3-H2}$ = 4.0Hz, 1H, H-3), 5.29 (t, $^3J_{H4-H3}$ = $^3J_{H4-H5}$ = 9.7Hz, 1H, H-4), 5.50 (d, $^3J_{H1-H2}$ = 2.7Hz, 1H, H-1) ppm

HRMS : calculated mass: 477.0372, found: 477.0381

1,2-*O*-(1-(2-bromoethoxy)ethylidene)- 3,4,6-tri-*O*-benzyl-α-D-mannopyranose (7)

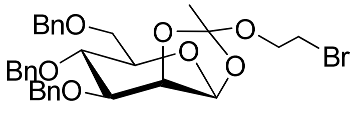

7

Compound **6** (4.79 g, 10.6 mmol) was dissolved in THF (22 mL). Then, NaOH 1N (43 mL) and methanol (2 mL) were added. The solution was stirred at RT for 16 h. AcOEt (250 mL) was added and the aqueous layer was extracted with AcOEt (3x200 mL). The organic layers were combined, dried ($MgSO_4$) and concentrated. The residue was purified by flash chromatography (CH_2Cl_2/MeOH 0% to 10%) to give the deacetylated compound in 82% yield.

The deacetylated compound (18.12 g, 55 mmol) was dissolved in DMF (141 mL). Benzyl bromide (26 mL, 220 mmol) was added. The solution was cooled to 0°C and NaH 60% dispersion in oil (5.5 g, 165 mmol) was added. After 16 h of stirring at RT, the solution was diluted with Et_2O (330 mL) and water (250 mL). The aqueous layer was extracted with Et_2O (2 x 700 mL). The combined organic layers were washed with water (8 x 300 mL), dried ($MgSO_4$) and concentrated. The residue was purified by silica gel (CH_2Cl_2/ Et_2O 20% to 100%) to give compound **7** in 48% yield.

^{1}H NMR (400 MHz, $CDCl_3$, 298K) δ 1.75 (s, 3H, CH_3), 3.42-3.49 (m, 3H, H-5, C<u>H</u>$_2$Br), 3.69-3.77 (m, 3H, H-6, H-6′, H-3), 3.78-3.84 (m, 2H, O-C<u>H</u>$_2$-CH_2), 3.92 (t, 1H, $^3J_{H3-H4}$ =$^3J_{H4-H5}$ = 9.2Hz, H-4), 4.44 (dd, 1H, $^3J_{H2-H1}$ = 2.8Hz, $^3J_{H2-H3}$ = 4.0Hz, H-2), 4.56 (d, 1H, $^2J_{H-H}$ = 12.1Hz, C<u>H</u>$_2$-Ph), 4.62 (d, 1H, $^2J_{H-H}$ = 12.1Hz, C<u>H</u>$_2$-Ph), 4.63 (d, 1H, $^2J_{H-H}$ = 10.8Hz, C<u>H</u>$_2$-Ph), 4.80 (s, 1H, C<u>H</u>$_2$-Ph), 4.81 (s, 1H, C<u>H</u>$_2$-Ph), 4.91 (d, 1H, $^2J_{H-H}$ = 10.8Hz, C<u>H</u>$_2$-Ph), 5.36 (d, 1H, $^3J_{H1-H2}$ = 2.8Hz, H-1), 7.23-7.42 (m, 15H, Ar) ppm
^{13}C NMR (100 MHz, $CDCl_3$, 298K) δ 22.4 (1C, <u>C</u>H$_3$), 30.4 (1C, <u>C</u>H$_2$-Br), 63.0 (1C, O-<u>C</u>H$_2$-CH_2), 69.1 (1C, C-6), 72.7 (1C, <u>C</u>H$_2$-Ph), 73.5 (1C, <u>C</u>H$_2$-Ph), 74.3 (1C, C-4), 74.4 (1C, C-5), 75.4 (1C, <u>C</u>H$_2$-Ph), 77.0 (1C, C-2), 79.0 (1C, C-3), 97.7 (1C, C-1), 123.7-138.3 (18C, Ar), 123.7 (C_{IV}) ppm

MS, ESI$^+$ *m/z* : 621 [**M**+Na]$^+$

2-bromoethyl 2-*O*-acetyl-3,4,6-tri-*O*-benzyl-*α*-D-mannopyranoside (8)

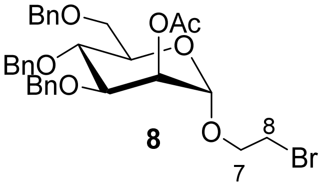

8

Compound **7** (1 g, 1.67 mmol) was dissolved in CH_2Cl_2 (20 mL). 2-bromoethanol (236 µL, 3.37 mmol) was added. After 10 min of stirring at RT, boron trifluoride diethyl etherate (211 µL, 1.67 mmol) was added. The solution was stirred at RT for 16 h. The reaction was quenched with a saturated solution of $NaHCO_3$ (50 mL). The aqueous layer was extracted with CH_2Cl_2 (3 x 50 mL). The combined organic layers were washed with brine (100 mL), dried ($MgSO_4$) and concentrated. The crude was purified by flash chromatography (cyclohexane/Et_2O 0% to 30%) to give the compound **8** in 75% yield.

^{1}H NMR (300 MHz, CDCl$_3$, 298 K): δ 2.15 (s, 3H, CH$_3$CO), 3.46 (t, 2H, ^{3}J = 6.1Hz, H-8), 3.72 (t, 1H, $^{3}J_{H\text{-}H}$ = 9.0Hz, H-4), 4.05-3.77 (m, 6H, H-3, H-5, H-6, H-6′, H-7, H-7′), 4.75-4.45 (m, 5H, C<u>H</u>$_2$-Ph), 4.85 (d, 1H, $^{2}J_{H\text{-}H}$ = 10.7 Hz, C<u>H</u>$_2$-Ph), 4.90 (d, 1H, $^{3}J_{H1\text{-}H2}$ = 1.7 Hz, H-1), 5.39 (dd, 1H, $^{3}J_{H2\text{-}H3}$ = 3.2Hz, $^{3}J_{H1\text{-}H2}$ = 1.7Hz, H-2), 7.17-7.14 (m, 2H, Ar), 7.38-7.27 (m, 13H, Ar) ppm

^{13}C NMR (100 MHz, CDCl$_3$, 298K) δ 21.2 (1C, <u>C</u>H$_3$-CO), 30.0 (1C, C-8), 68.1 (1C, C-7), 68.8 (1C, C-2), 69.0 (1C, C-6), 72.0 (2C, C-4 and <u>C</u>H$_2$-Ph), 73.7 (1C, <u>C</u>H$_2$-Ph), 74.3 (1C, C-5), 75.3 (3C, <u>C</u>H$_2$-Ph), 78.2 (1C, C-3), 98.2 (1C, C-1), 127.7-128.5 (15C, Ar), 138.0-135.5 (3C, Cq arom), 170.6 (1C, C=O) ppm

MS, ESI$^+$ *m/z* : 605.2 [**M**+Na]$^+$

2-bromoethyl 3,4,6-tri-*O*-benzyl-*α*-D-mannopyranoside (9)

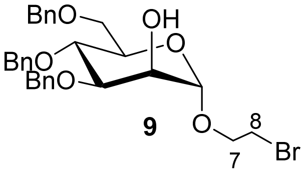

Compound **8** (750 mg, 1.25 mmol) was dissolved in THF (1.6 mL). Aqueous solution of NaOH 1N was added (2.5 mL, 2.5 mmol) and the solution was stirred at RT for 21 h. The solution was neutralized with HCl 1N. CH$_2$Cl$_2$ (40 mL) was added and the aqueous layer was extracted with CH$_2$Cl$_2$ (3 x 40 mL). The combined organic layers were dried (MgSO$_4$) and concentrated. The residue was purified by flash chromatography (cyclohexane/ Et$_2$O 0% to 30%) to give the compound **9** in 82% yield.

^{1}H NMR (400 MHz, CDCl$_3$, 298 K): δ 3.49 (td, 2H, $^{3}J_{H\text{-}H}$ = 6.0Hz, $^{3}J_{H\text{-}H}$ = 1.3Hz, H-8), 3.79-3.70 (m, 2H, H-4, H-5), 3.94-3.79 (m, 4H, H-3, H-6, H-6′, H-7), 3.99 (dt, 1H, $^{3}J_{H7'\text{-}H8}$ = $^{3}J_{H7'\text{-}H8'}$ = 6.0Hz, $^{2}J_{H7'\text{-}H7}$ = 10.6Hz, H-7′), 4.09 (dd, 1H, $^{3}J_{H2\text{-}H1}$ = 1.6Hz, $^{3}J_{H2\text{-}H3}$ = 3.0Hz, H-2), 4.53 (d, 1H, ^{2}J = 10.8Hz, C<u>H</u>$_2$-Ph), 4.55 (d, 1H, ^{2}J = 12.2Hz, C<u>H</u>$_2$-Ph), 4.66 (d, 1H, ^{2}J = 12.2Hz, C<u>H</u>$_2$-Ph), 4.72 (s, 2H, C<u>H</u>$_2$-Ph), 4.84 (d, 1H, ^{2}J = 10.8Hz, C<u>H</u>$_2$-Ph), 4.98 (d, 1H, $^{3}J_{H\text{-}H}$ = 1.6Hz, H-1), 7.23-7.16 (m, 2H, Ar), 7.41-7.28 (m, 13H, Ar) ppm

^{13}C NMR (100 MHz, CDCl$_3$, 298K) δ 30.7 (1C, C-8), 68.3 (1C, C-7), 68.8 (1C, C-2), 69.6 (1C, C-6), 72.1 (1C, C-4), 72.7 (1C, <u>C</u>H$_2$-Ph), 74.1 (1C, <u>C</u>H$_2$-Ph), 74.8 (1C, C-5), 75.7 (1C, <u>C</u>H$_2$-Ph), 80.6 (1C, C-3), 100.0 (1C, C-1), 127.8-128.8 (15C, Ar), 138.1-138.4 (3C, Cq arom) ppm

MS, ESI$^+$ *m/z* : 579.1 [**M**+Na]$^+$

2-bromoethyl 2,3,4,6-tetra-*O*-acetyl-*α*-D-mannopyranosyl-(1 2)-3,4,6-tri-*O*-benzyl-*α*-D-mannopyranoside (11)

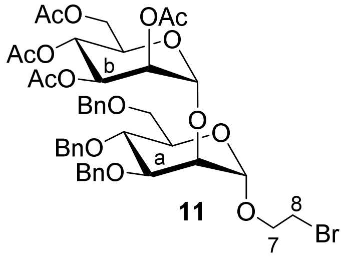

11

Compound **9** (430 mg, 0.77 mmol) and 2,3,4,6-Tetra-O-acetyl-α-D-mannopyranosyl trichloroacetimidate **10** (860 mg, 1.75 mmol) were dissolved in CH_2Cl_2 (15 mL) with previously activated 4 Å molecular sieves. The solution was stirred at RT for 30 min and then cooled to -30°C. TMSOTf (167 µL, 0.924 mmol) was added dropwise and the solution was stirred at -30°C for 1 h 15. The reaction was quenched with pyridine (3.5 mL) and filtered over celite. The filtrate was concentrated. The crude was purified by flash chromatography (cyclohexane/ Et_2O 30%) to give the compound **11** in 64% yield.

^{1}H NMR (400 MHz, $CDCl_3$, 298K) δ 2.00 (s, 3H, CH_3-CO), 2.01 (s, 3H, CH_3-CO), 2.10 (s, 3H, CH_3-CO), 2.11 (s, 3H, CH_3-CO), 3.42-3.53 (m, 2H, CH_2-Br), 3.70-3.73 (m, 2H, H-6a, H-6a'), 3.75-3.82 (m, 1H, CH_2-O), 3.84-3.85 (m, 1H, H-5a), 3.87 (t, $^3J_{H3a-H4a}$ =$^3J_{H4a-H5a}$= 9.4Hz, 1H, H-4a), 3.93 (dd, $^3J_{H3a-H2a}$= 2.2Hz, $^3J_{H3a-H4a}$= 9.4Hz, 1H, H-3a), 3.96-4.03 (m, 2H, H-2a, CH_2-O), 4.10 (dd, $^3J_{H6'b-H5b}$= 1.8Hz, $^2J_{H6'b-H6b}$= 11.9Hz, 1H, H-6b'), 4.18 (ddd, $^3J_{H5b-H6'b}$= 1.8Hz, $^3J_{H5b-H6b}$= 5.2Hz, $^3J_{H5b-H4b}$= 10.0Hz, 1H, H-5b), 4.25 (dd, $^3J_{H6b-H5b}$= 5.2Hz, $^2J_{H6b-H6'b}$= 11.9Hz, 1H, H-6b), 4.51 (d, 1H, $^2J_{H-H}$= 11.1Hz, C$\underline{H_2}$-Ph), 4.57 (d, 1H, $^2J_{H-H}$= 12.2Hz, C$\underline{H_2}$-Ph), 4.62 (d, 1H, $^2J_{H-H}$= 5.5Hz, C$\underline{H_2}$-Ph), 4.65 (d, 1H, $^2J_{H-H}$= 5.5Hz, C$\underline{H_2}$-Ph), 4.74 (d, 1H, $^2J_{H-H}$= 12.2Hz, C$\underline{H_2}$-Ph), 4.82 (d, 1H, $^2J_{H-H}$= 11.1Hz, C$\underline{H_2}$-Ph), 4.96 (d, 1H, $^3J_{H2a-H1a}$= 1.8Hz, H-1a), 4.99 (d, 1H, $^3J_{H2b-H1b}$= 1.8Hz, H-1b), 5.26 (t, $^3J_{H4b-H3b}$ =$^3J_{H4b-H5b}$= 10.0Hz, 1H, H-4b), 5.41 (dd, $^3J_{H3b-H2b}$= 3.2Hz, $^3J_{H3b-H4b}$= 10.0Hz, 1H, H-3b), 5.45 (dd, 1H, $^3J_{H2b-H1b}$= 1.8Hz, $^3J_{H2b-H3b}$= 3.2Hz, H-2b), 7.16-7.38 (m, 15H, Ar) ppm

^{13}C NMR (100 MHz, $CDCl_3$, 298K) δ 20.9 (1C, $\underline{C}H_3$-CO), 20.9 (1C, $\underline{C}H_3$-CO), 21.0 (1C, $\underline{C}H_3$-CO), 21.1 (1C, $\underline{C}H_3$-CO), 30.4 (1C, CH_2-Br), 62.9 (1C, C-6b), 66.5 (1C, C-4b), 68.0 (1C, CH_2-O), 69.1 (1C, C-5b), 69.3 (1C, C-3b), 69.3 (1C, C-6a), 69.6 (1C, C-2b), 72.4 (1C, C-5a), 72.7 (1C, Ph-$\underline{C}H_2$), 73.4 (1C, Ph-$\underline{C}H_2$), 74.9 (1C, C-4a), 75.4 (1C, Ph-$\underline{C}H_2$), 76.2 (1C, C-2a), 79.5 (1C, C-3a), 99.1 (1C, C-1a), 99.4 (1C, C-1b), 127.7-128.6 (15C, Ar), 138.4-138.5 (3C, Ar Cq), 169.8 (1C, C=O), 169.8 (1C, C=O), 169.9 (1C, C=O), 170.7 (1C, C=O) ppm

MS, ESI$^+$ *m/z* : 909.4 [**M**+Na]$^+$

2-bromoethyl 2,3,4,6-tetra-*O*-trimethysilyl-α-D-mannopyranosyl-(1 2)-3,4,6-tri-*O*-benzyl-α-D-mannopyranoside (12)

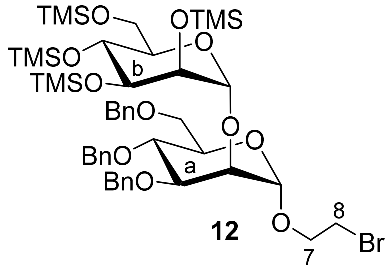

12

Compound **11** (5.84 g, 6.58 mmol) was dissolved in THF (16 mL). NaOH 1N (33 mL, 32.9 mmol) and methanol (1.5 mL) were added. The solution was stirred at RT for 18 h, neutralized with HCl 1N and concentrated. The crude was purified on silica gel to give the compound in 73% yield.

The deacetylated compound (3.4 g, 4.72 mmol) was dissolved in CH_2Cl_2 (24 mL) and triethylamine (19 mL, 141.6 mmol). The solution was cooled to 0°C and trimethylsilyl chloride (4.8 mL, 37.8 mmol) was added dropwise. After 18 h of stirring at RT, the solution was concentrated. The residue was taken in cyclohexane and filtered over celite. The filtrate was concentrated to give the compound **12** directly used for next step.

^{1}H NMR (600 MHz, $CDCl_3$, 298K) δ 0.06 (s, 9H, C<u>H</u>$_3$-Si) , 0.13 (s, 9H, C<u>H</u>$_3$-Si), 0.13 (s, 9H, C<u>H</u>$_3$-Si), 0.15 (s, 9H, C<u>H</u>$_3$-Si), 3.30-3.41 (m, 2H, H-8), 3.55-3.60 (m, 1H, H-7), 3.60-3.71 (m, 4H, H-5, H-6′, H-6, H-6′), 3.75-3.84 (m, 6H, H-6, H-7, H-4, H-4, H-3, H-5), 3.87 (dd, $^{3}J_{H2-H1}$ = 1.9Hz, $^{3}J_{H2-H3}$ = 2.3Hz, 1H, H-2), 3.90 (dd, $^{3}J_{H3-H2}$ = 2.8Hz, $^{3}J_{H3-H4}$ = 9.2Hz, 1H, H-3), 4.07 (dd, $^{3}J_{H2-H1}$ = 1.6Hz, $^{3}J_{H2-H3}$ = 2.8Hz, 1H, H-2), 4.50 (d, $^{2}J_{H-H}$ = 10.7Hz, 1H, Ph-C<u>H</u>$_2$-O), 4.56 (d, $^{2}J_{H-H}$ = 12.4Hz, 1H, Ph-C<u>H</u>$_2$-O), 4.62 (d, $^{2}J_{H-H}$ = 12.4Hz, 1H, Ph-C<u>H</u>$_2$-O), 4.67 (d, $^{2}J_{H-H}$ = 11.5Hz, 1H, Ph-C<u>H</u>$_2$-O), 4.69 (d, $^{2}J_{H-H}$ = 11.5Hz, 1H, Ph-C<u>H</u>$_2$-O), 4.80 (d, $^{2}J_{H-H}$ = 10.7Hz, 1H, Ph-C<u>H</u>$_2$-O), 4.86 (d, $^{3}J_{H1-H2}$ = 1.9Hz, 1H, H-1), 4.96 (d, $^{3}J_{H1-H2}$ = 1.6Hz, 1H, H-1), 7.16-7.35 (m, 15H, Ar) ppm
^{13}C NMR (150 MHz, $CDCl_3$, 298K) δ 0.0 (3C, <u>C</u>H$_3$-Si), 0.5 (3C, <u>C</u>H$_3$-Si), 0.6 (3C, <u>C</u>H$_3$-Si), 0.9 (3C, <u>C</u>H$_3$-Si), 30.5 (1C, C-8), 62.9 (1C, C-6), 67.8 (1C, C-7), 68.5 (1C), 69.3 (1C, C-6), 72.2 (1C), 72.3 (1C, Ph-<u>C</u>H$_2$-O), 73.4 (s, 1C, Ph-<u>C</u>H$_2$-O), 73.6 (1C, C-2), 74.1 (1C, C-2), 74.9 (1C, C-4), 75.2 (1C, Ph-<u>C</u>H$_2$-O), 75.6 (1C, C-5), 79.9 (1C, C-3), 99.5 (1C, C-1), 102.5 (1C, C-1), 127.2-128.8 (15C, Ar), 138.5-138.6 (3C, Ar_q) ppm

2-bromoethyl 2,3,4-tri-*O*-trimethysilyl-*α*-D-mannopyranosyl-(1 2)-3,4,6-tri-*O*-benzyl-*α*-D-mannopyranoside (13)

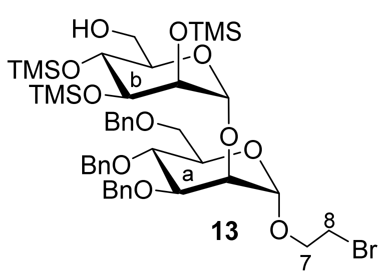

13

The tetrasilylated compound **12** (4.58 g, 4.72 mmol) was dissolved in methanol (65 mL). A solution of K_2CO_3 (6.5 mg, 0.0472 mmol) in methanol (10 mL) was added dropwise. After 1 h 45 of stirring at RT, the solution was diluted with CH_2Cl_2 (200 mL) and washed with brine (125 mL). The aqueous layer was extracted with CH_2Cl_2 (260 mL). The combined organic layers were dried ($MgSO_4$) and concentrated. The crude was purified on silica gel (cyclohexane/Et_2O 30% to 70%) to give the title compound **13** in 81% yield over 2 steps.

^{1}H NMR (600 MHz, $CDCl_3$, 298K) δ 0.08 (s, 9H, C<u>H</u>$_3$-Si), 0.14 (s, 9H, C<u>H</u>$_3$-Si), 0.16 (s, 9H, C<u>H</u>$_3$-Si), 2.0 (s, 1H, O<u>H</u>), 3.44-3.50 (m, 2H, H-8), 3.65 (dd, $^3J_{H6'-H5}$ = 1.8Hz, $^2J_{H6'-H6}$ = 10.8Hz, 1H, H-6'), 3.67-3.96 (m, 12H, H-6', 2H-6, 2H-7, 2H-5, 2H-3, 2H-4, H-2), 4.01 (dd, $^3J_{H2-H1}$ = 1.6Hz, $^3J_{H2-H3}$ = 3.0Hz, 1H, H-2), 4.49 (d, $^2J_{H-H}$ = 10.8Hz, 1H, Ph-C<u>H</u>$_2$-O), 4.55 (d, $^2J_{H-H}$ = 12.3Hz, 1H, Ph-C<u>H</u>$_2$-O), 4.62 (d, $^2J_{H-H}$ = 12.3Hz, 1H, Ph-C<u>H</u>$_2$-O), 4.68 (d, $^2J_{H-H}$ = 11.5Hz, 1H, Ph-C<u>H</u>$_2$-O), 4.71 (d, $^2J_{H-H}$ = 11.5Hz, 1H, Ph-C<u>H</u>$_2$-O), 4.79 (d, $^2J_{H-H}$ = 10.8Hz, 1H, Ph-C<u>H</u>$_2$-O), 4.86 (d, $^3J_{H1-H2}$ = 1.8Hz, 1H, H-1), 4.94 (d, $^3J_{H1-H2}$ = 1.6Hz, 1H, H-1), 7.15-7.36 (m, 15H, Ar) ppm
^{13}C NMR (150 MHz, $CDCl_3$, 298K) δ 0.4 (3C, <u>C</u>H$_3$-Si), 0.7 (3C, <u>C</u>H$_3$-Si), 0.8 (3C, <u>C</u>H$_3$-Si), 30.5 (1C, C-8), 62.4 (1C, C-6), 67.8 (1C, C-7), 68.1 (1C), 69.1 (1C, C-6), 72.2 (1C), 72.3 (1C), 72.7 (1C, Ph-<u>C</u>H$_2$-O), 73.4 (s, 1C, Ph-<u>C</u>H$_2$-O), 73.8 (1C, C-2), 74.8 (2C, C-2 + 1C), 75.0 (1C, C-4), 75.2 (1C, Ph-<u>C</u>H$_2$-O), 80.0 (1C, C-3), 99.3 (1C, C-1), 102.6 (1C, C-1), 127.6-128.6 (15C, Ar), 138.4-138.5 (3C, Ar$_q$) ppm

MS, ESI$^+$ *m/z* : 959.5 [**M**+Na]$^+$

2-bromoethyl (6E)-6,7-dideoxy-7-ethoxycarbonyl-2,3,4-tri-*O*-trimethylsilyl-α-D-*manno*-hept-6-enopyranosyl-(1 2)-3,4,6-tri-*O*-benzyl-α-D-mannopyranoside (14)

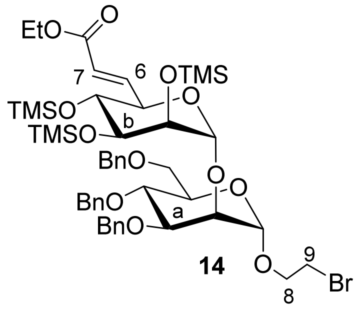

14

Compound **13** (1 g, 1.07 mmol) was dissolved in CH_2Cl_2 (24 mL). A solution of Dess-Martin periodinane 3M in CH_2Cl_2 (5.4 mL, 1.6 mmol) was added dropwise. The solution was stirred at RT for 4 h and then diluted with Et_2O (120 mL). A saturated solution of $NaHCO_3$ (25 mL) and sodium thiosulfate (3.9 g) were added. The solution was stirred at RT for 5 min. The aqueous layer was extracted with Et_2O (3 x 125 mL). The combined organic layers were dried ($MgSO_4$) and concentrated.

To NaH 60% (144 mg, 4.28 mmol) dissolved in THF (10 mL) was added dropwise triethylphosphonoacetate (637 μL, 3.21 mmol). After 45 min of stirring at RT, the solution

was added to the aldehyde (1.065 g, 1.07 mmol) dissolved in THF (20 mL). The solution was stirred at RT for 14 h and then diluted with CH_2Cl_2 (175 mL) and washed with brine (4 x 50 mL). The aqueous layer was extracted with CH_2Cl_2 (125 mL). The combined organic layers were dried ($MgSO_4$) and concentrated. The crude was purified by flash chromatography (cyclohexane/Et_2O 20%) to give the compound **14** in 75% over 2 steps.

^{1}H NMR (400 MHz, MeOD, 298K) δ 0.09 (s, 9H, $C\underline{H}_3$-Si), 0.10 (s, 9H, $C\underline{H}_3$-Si), 0.17 (s, 9H, $C\underline{H}_3$-Si), 1.28 (t, 3H, $^3J_{H\text{-}H}$ = 7.1Hz, $C\underline{H}_3$-CH_2-O), 3.52 (t, 2H, $^3J_{H\text{-}H}$ = 5.7Hz, H-9), 3.59 (dd, $^3J_{H6'a\text{-}H5a}$ = 1.8Hz, $^2J_{H6'a\text{-}H6a}$ = 10.9Hz, 1H, H-6'a), 3.66 (dd, $^3J_{H6a\text{-}H5a}$ = 4.3Hz, $^2J_{H6a\text{-}H6'a}$ = 10.9Hz, 1H, H-6a), 3.71 (t, $^3J_{H4b\text{-}H3b}$ = 9.2Hz, 1H, H-4b), 3.73-3.80 (m, 2H, H-8', H-5a), 3.89-3.94 (m, 4H, H-8, H-3a, H-3b, H-4a), 3.96-3.97 (m, 1H, H-2), 4.05-4.06 (m, 1H, H-2), 4.16-4.21 (m, 2H, CH_3-$C\underline{H}_2$-O), 4.35 (ddd, $^4J_{H5b\text{-}H7b}$ = 1.5Hz, $^3J_{H5b\text{-}H6b}$ = 5.0 Hz, $^3J_{H5b\text{-}H4b}$ = 9.2Hz, 1H, H-5b), 4.53 (d, $^2J_{H\text{-}H}$ = 12.5Hz, 1H, Ph-$C\underline{H}_2$-O), 4.55 (d, $^2J_{H\text{-}H}$ = 11.0Hz, 1H, Ph-$C\underline{H}_2$-O), 4.59 (d, $^2J_{H\text{-}H}$ = 12.5Hz, 1H, Ph-$C\underline{H}_2$-O), 4.65 (d, $^2J_{H\text{-}H}$ = 11.5Hz, 1H, Ph-$C\underline{H}_2$-O), 4.69 (d, $^2J_{H\text{-}H}$ = 11.5Hz, 1H, Ph-$C\underline{H}_2$-O), 4.79 (d, $^2J_{H\text{-}H}$ = 11.0Hz, 1H, Ph-$C\underline{H}_2$-O), 4.90 (d, 1H, $^3J_{H1\text{-}H2}$ = 1.8Hz, H-1), 4.91 (d, 1H, $^3J_{H1\text{-}H2}$ = 1.8Hz, H-1), 6.10 (dd, 1H, $^4J_{H7b\text{-}H5b}$ = 1.5Hz, $^3J_{H7b\text{-}H6b}$ = 15.7Hz, H-7b), 7.01 (dd, $^3J_{H6b\text{-}H5b}$ = 5.0Hz, $^3J_{H6b\text{-}H7b}$ = 15.7Hz, 1H, H-6b), 7.17-7.37 (m, 15H, Ar) ppm
^{13}C NMR (100 MHz, MeOD, 298K) δ 0.6 (3C, $\underline{C}H_3$-Si), 0.7 (3C, $\underline{C}H_3$-Si), 0.9 (3C, $\underline{C}H_3$-Si), 14.6 (1C, $\underline{C}H_3$-CH_2-O), 31.6 (1C, C-10), 61.6 (1C, CH_3-$\underline{C}H_2$-O), 69.1 (1C, C-9), 69.7 (1C, C-6a), 72.8 (1C, C-4b), 73.2 (1C, C-5a), 73.3 (1C, Ph-$\underline{C}H_2$-O), 73.8 (1C), 74.3 (1C, Ph-$\underline{C}H_2$-O), 74.5 (1C, C-5b), 75.0 (1C, C-2), 75.8 (1C), 76.1 (1C, Ph-$\underline{C}H_2$-O), 76.6 (1C, C-2), 80.9 (1C), 100.4 (1C, C-1), 103.8 (1C, C-1), 122.7 (1C, C-7b), 128.7-129.4 (15C, Ar), 139.6-139.8 (3C, Ar_q), 146.9 (1C, C-6b), 167.8 (1C, C-8) ppm

MS, ESI$^+$ *m/z* : 1027 [**M**+Na]$^+$

2-azidoethyl 6,7-dideoxy-7-ethoxycarbonyl-*α*-D-*manno*-heptopyranosyl-(1 2)-*α*-D-mannopyranoside (15)

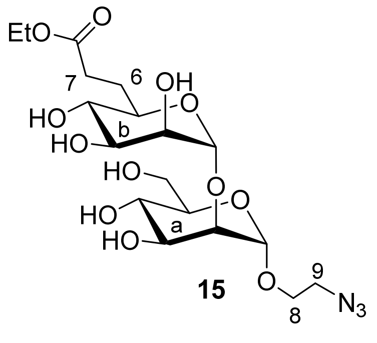

Compound **14** (795 mg, 0.79 mmol) was dissolved in methanol (8 mL). 10% palladium on charcoal (160 mg) was added. Triethylsilane (1.3 mL, 7.9 mmol) was added dropwise over 1 h. The solution was then stirred at RT for 20 min and filtered over celite. The filtrate was concentrated.

The residue (440 mg, 0.85 mmol) was dissolved in DMF (3.5 mL). NaN_3 (66 mg, 1.02 mmol) was added. The solution was stirred at RT for 5 days and concentrated. The crude was purified by flash chromatography (AcOEt/MeOH 10% to 20%) to give the compound **15** in 99% yield.

^{1}H NMR (600 MHz, MeOD, 298K) δ 1.26 (t, 3H, $^{3}J_{H-H}$ = 7.2Hz, C<u>H</u>$_3$-CH$_2$-O), 1.78 (dddd, $^{3}J_{H6'b-H5b}$ = 5.9Hz, $^{3}J_{H6'b-H7b}$ = 6.2Hz, $^{3}J_{H6'b-H7'b}$ = 8.3Hz, $^{2}J_{H6'b-H6b}$ = 14.2Hz, 1H, H-6'b), 2.19 (dddd, $^{3}J_{H6b-H5b}$ = 2.3Hz, $^{3}J_{H6b-H7'b}$ = 6.9Hz, $^{3}J_{H6b-H7b}$ = 8.7Hz, $^{2}J_{H6b-H6'b}$ = 14.2Hz, 1H, H-6b), 2.44 (ddd, $^{3}J_{H7'b-H6b}$ = 6.9Hz, $^{3}J_{H7'b-H6'b}$ = 8.3Hz, $^{2}J_{H7'b-H7b}$ = 15.8Hz, 1H, H-7'b), 2.52 (ddd, $^{3}J_{H7b-H6'b}$ = 6.2Hz, $^{3}J_{H7b-H6b}$ = 8.7Hz, $^{2}J_{H7b-H7'b}$ = 15.8Hz, 1H, H-7b), 3.43-3.46 (m, 2H, H-9), 3.44 (t, $^{3}J_{H4b-H3b}$ = 9.5Hz, 1H, H-4b), 3.56 (ddd, $^{3}J_{H5b-H6b}$ = 2.3Hz, $^{3}J_{H5b-H6'b}$ = 5.9Hz, $^{3}J_{H5b-H4b}$ = 9.5Hz, 1H, H-5b), 3.61 (t, $^{3}J_{H4a-H3a}$ = 9.5Hz, 1H, H-4a), 3.60-3.62 (m, 2H, H-5a, H-3b), 3.68-3.69 (m, 2H, H-8', H-6'a), 3.84-3.86 (m, 2H, H-6a, H-3a), 3.87 (dd, $^{3}J_{H2a-H1a}$ = 1.8Hz, $^{3}J_{H2a-H3a}$ = 3.5Hz, 1H, H-2a), 3.92 (ddd, $^{3}J_{H9-H10}$ = 4.4Hz, $^{3}J_{H9-H10'}$ = 5.7Hz, $^{3}J_{H9-H9'}$ = 11.0Hz, 1H, H-8), 3.96 (dd, $^{3}J_{H2b-H1b}$ = 1.8Hz, $^{3}J_{H2b-H3b}$ = 3.3Hz, 1H, H-2b), 4.09-4.18 (m, 2H, CH$_3$-C<u>H</u>$_2$-O), 4.98 (d, $^{3}J_{H1a-H2a}$ = 1.8Hz, 1H, H-1a), 4.99 (d, $^{3}J_{H1b-H2b}$ = 1.8Hz, 1H, H-1b) ppm

^{13}C NMR (150 MHz, MeOD, 298K) δ 14.5 (1C, <u>C</u>H$_3$-CH$_2$-O), 28.3 (1C, C-6b), 31.4 (1C, C-7b), 51.8 (1C, C-10), 61.5 (1C, CH$_3$-<u>C</u>H$_2$-O), 63.0 (1C, C-6a), 67.7 (1C, C-9), 68.9 (1C, C-4a), 71.8 (1C, C-2b), 72.2 (1C, C-4b), 72.3 (1C, C-3b), 72.3 (1C, C-3a), 73.1 (1C, C-5a), 75.1 (1C, C-5b), 79.2 (1C, C-2a), 100.5 (1C, C-1a), 103.8 (1C, C-1b), 175.6 (1C, C-8) ppm

MS, ESI^{+} *m/z* : 482,4 [**M**+H]$^{+}$

2-aminoethyl 6,7-dideoxy-7-hydroxycarbonyl-*α*-D-*manno*-heptopyranosyl-(1 2)-*α*-D-mannopyranoside (16)

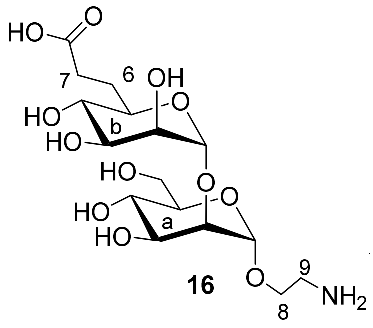

Compound **15** (377 mg, 0.78 mmol) was dissolved in NaOH 1N (932 μL, 0.932 mmol). The solution was strirred at RT for 20 h and then concentrated. The residue was purified on silica gel (isopropanol/NH_4OH 35%/water 6:3:1) to give the carboxylic acid compound in 86% yield.

The carboxylic acid compound (316 mg, 0.67 mmol) was dissolved in a mixture of methanol/water (2:1) (15 mL). 10% palladium on charcoal (32 mg) was added.

Triethylsilane (536 µL, 3.36 mmol) was added dropwise over 40 min. After 45 min stirring at RT, triethylsilane (536 µL, 3.36 mmol) was added dropwise over 30 min. The suspension was stirred at RT for 10 min and then filtered over celite. The filtrate was concentrated. The residue was dissolved in methanol/water (2:1) (3 mL). 10% palladium on charcoal (60 mg) was added. Triethylsilane (1.07 mL, 6.72 mmol) was added dropwise over 20 min. The suspension was stirred at RT for 30 min and then filtered over celite. The filtrate was concentrated to afford compound **16** in 97% yield.

^{1}H NMR (600 MHz, D_2O, 298K) δ 1.66 (dddd, ${}^{3}J_{H6'b-H7b}$ = 5.9Hz, ${}^{3}J_{H6'b-H7'b}$ = 6.9Hz, ${}^{3}J_{H6'b-H5b}$ = 9.7Hz, ${}^{2}J_{H6'b-H6b}$ = 14.3Hz, 1H, H-6′b), 2.16 (dtd, ${}^{3}J_{H6b-H5b}$ = 2.2Hz, ${}^{3}J_{H6b-H7b}$ = ${}^{3}J_{H6b-H7'b}$ = 7.9Hz, ${}^{2}J_{H6b-H6'b}$ = 14.3Hz, 1H, H-6b), 2.33 (ddd, ${}^{3}J_{H7'b-H6'b}$ = 6.9Hz, ${}^{3}J_{H7'b-H6b}$ = 7.9Hz, ${}^{2}J_{H7'b-H7b}$ = 15.5Hz, 1H, H-7′b), 2.39 (ddd, ${}^{3}J_{H7b-H6'b}$ = 5.9Hz, ${}^{3}J_{H7b-H6b}$ = 7.9Hz, ${}^{2}J_{H7b-H7'b}$ = 15.5Hz, 1H, H-7b), 3.23 (ddd, ${}^{3}J_{H10'-H9}$ = 3.7Hz, ${}^{3}J_{H10'-H9'}$ = 7.6Hz, ${}^{2}J_{H10'-H10}$ = 13.7Hz, 1H, H-9′), 3.29 (ddd, ${}^{3}J_{H10-H9'}$ = 3.7Hz, ${}^{3}J_{H10-H9}$ = 5.3Hz, ${}^{2}J_{H10-H10'}$ = 13.7Hz, 1H, H-9), 3.48 (t, 1H, ${}^{3}J_{H4b-H3b}$ = 9.7Hz, H-4b), 3.58-3.61 (m, 1H, H-5b), 3.60-3.62 (m, 1H, H-5a), 3.68 (t, ${}^{3}J_{H4a-H3a}$ = 9.4Hz, 1H, H-4a), 3.70-3.76 (m, 2H, H-6′a, H-8′), 3.81 (dd, ${}^{3}J_{H3b-H2b}$ = 3.4Hz, ${}^{3}J_{H3b-H4b}$ = 9.7Hz, 1H, H-3b), 3.89 (dd, ${}^{3}J_{H6a-H5a}$ = 2.1Hz, ${}^{2}J_{H6a-H6'a}$ = 12.3Hz, 1H, H-6a), 3.95 (dd, ${}^{3}J_{H3a-H2a}$ = 3.3Hz, ${}^{3}J_{H3a-H4a}$ = 9.4Hz, 1H, H-3a), 3.98 (ddd, ${}^{3}J_{H9-H10'}$ = 3.7Hz, ${}^{3}J_{H9-H10}$ = 5.3Hz, ${}^{2}J_{H9-H9'}$ = 11.4Hz, 1H, H-8′), 4.03-4.05 (m, 2H, H-2b, H-2a), 4.98 (d, 1H, ${}^{3}J_{H1a-H2a}$ = 1.6Hz, H-1a), 5.05 (d, 1H, ${}^{3}J_{H1b-H2b}$ = 1.7Hz, H-1b) ppm
^{13}C NMR (150 MHz, D_2O, 298K) δ 27.4 (1C, C-6b), 32.7 (1C, C-7b), 39.0 (1C, C-10), 60.9 (1C, C-6a), 63.5 (1C, C-9), 66.8 (1C, C-4a), 69.9 (1C, C-2b), 70.1 (1C, C-3b), 70.5 (1C, C-3a), 70.6 (1C, C-4b), 71.9 (1C, C-5a), 73.1 (1C, C-5b), 76.4 (1C, C-2a), 98.6 (1C, C-1a), 101.6 (s, 1C, C-1b), 182.0 (1C, C-8) ppm

HRMS : calculated mass: 428.1768, found: 428.1768

2-[N-(2-Ethoxy-3,4-dioxocyclobut-1-enyl)-aminoethyl 6,7-dideoxy-7-hydroxycarbonyl-α-D-*manno*-heptopyranosyl-(1 2)-α-D-mannopyranoside (17)

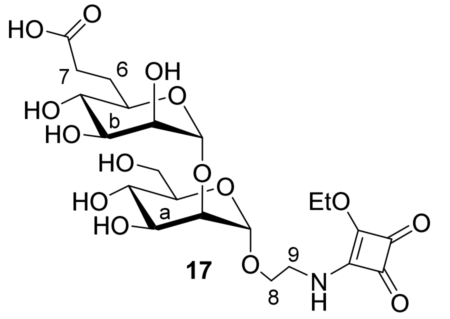

Diethylsquarate (45 µL, 0.307 mmol) was diluted in a mixture ethanol/water (2:1) (300 µL). Triethylamine (20 µL, 0.154 mmol) was added until pH = 8-9. Compound **16** (131 mg, 0.307 mmol) dissolved in a mixture ethanol/water (2:1) (1.7 mL) was added dropwise. The solution was stirred at RT for 2h30 while keeping pH=8-9 by addition of triethylamine and

then concentrated. The crude was purified on silica gel (AcOEt/MeOH 30% to 70%) to give the compound **17** in 70% yield.

$[\alpha]_D^{20}$ = +46,0° (c=5.10^{-3}M/H_2O)

^{1}H (600 MHz, D_2O, 353K) δ 1.97 (t, 1H, $^3J_{H\text{-}H}$ = 7.0Hz, $C\underline{H}_3$-CH_2-O), 2.28 (dtd, 1H, $^3J_{H6b'\text{-}H7b}$ = 6.1Hz, $^3J_{H6b'\text{-}H7b'}$ = 8.5Hz, $^2J_{H6b'\text{-}H6b}$ = 14.7Hz, H-6b′), 2.66 (dddd, 1H, $^3J_{H6b\text{-}H5b}$ = 2.7Hz, $^3J_{H6b\text{-}H7b'}$ = 7.0Hz, $^3J_{H6b\text{-}H7b}$ = 8.5Hz, $^2J_{H6b\text{-}H6b'}$ = 14.7Hz, H-6b), 2.89 (ddd, 1H, $^3J_{H7b'\text{-}H6b}$ = 7.0Hz, $^3J_{H7b'\text{-}H6b'}$ = 8.5Hz, $^2J_{H7b'\text{-}H7b}$ = 15.0Hz, H-7b′), 3.00 (ddd, 1H, $^3J_{H6b'\text{-}H7b}$ = 6.1Hz, $^3J_{H6b\text{-}H7b}$ = 8.5Hz, $^2J_{H7b'\text{-}H7b}$ = 15.0Hz, H-7b), 4.03 (t, 1H, $^3J_{H4b\text{-}H5b}$ = $^3J_{H4b\text{-}H3b}$ = 9.6Hz, H-4b), 4.06 (ddd, 1H, $^3J_{H5a\text{-}H4a}$ = 2.3Hz, $^3J_{H5a\text{-}H6a'}$ = 5.9Hz, $^3J_{H5a\text{-}H4a}$ = 9.6Hz, H5a), 4.15 (dt, 1H, $^3J_{H6b\text{-}H5b}$ = 2.7Hz, $^3J_{H4b\text{-}H5b}$ = 9.3Hz, H5b), 4.19 (t, 1H, $^3J_{H4a\text{-}H5a}$ = $^3J_{H4a\text{-}H3a}$ = 9.7Hz, H-4a), 4.23 (ddd, 1H, H-8), 4.26 (ddd, 1H, $^3J_{H6a'\text{-}H5a}$ = 5.9Hz, $^2J_{H6a'\text{-}H6a}$ = 12.3Hz, H-6a′), 4.26-4.30 (m, 2H, H-9), 4.31 (dd, 1H, $^3J_{H3b\text{-}H2b}$ = 3.5Hz, $^3J_{H3b\text{-}H4b}$ = 9.6Hz, H-3b), 4.33 (dd, 1H, $^3J_{H3a\text{-}H2a}$ = 3.0Hz, $^3J_{H3a\text{-}H4a}$ = 9.6Hz, H-3a), 4.37 (dd, 1H, $^3J_{H6a\text{-}H5a}$ = 2.3Hz, $^2J_{H6a\text{-}H6a'}$ = 12.3Hz, H-6a), 4.40 (ddd, 1H, H-8′), 4.42 (dd, 1H, $^3J_{H2a\text{-}H1a}$ = 1.1Hz, $^3J_{H2a\text{-}H3a}$ = 3.0Hz, H-2a), 4.56 (dd, 1H, $^3J_{H2b\text{-}H1b}$ = 1.1Hz, $^3J_{H2b\text{-}H3b}$ = 3.5Hz, H-2b), 5.27 (q, 2H, $^3J_{H\text{-}H}$ = 7.0Hz, CH_3-$C\underline{H}_2$-O), 5.48 (d, 1H, $^3J_{H1a\text{-}H2a}$ = 1.1Hz, H-1a) , 5.50 (d, 1H, $^3J_{H1b\text{-}H2b}$ = 1.1Hz, H-1b) ppm
^{13}C NMR (150 MHz, D_2O, 353K) δ 16.9 (1C, $\underline{C}H_3$-CH_2-O), 28.9 (1C, C-6b), 33.1 (1C, C-7b), 46.0 (1C, C-10), 63.1 (1C, C-6a), 68.7 (s, 1C, C-9), 69.0 (1C, C-4a), 72.0 (1C, C-2b), 72.3 (1C), 72.5 (2C), 72.7 (1C, CH_3-$\underline{C}H_2$-O), 74.0 (1C, C-5b), 75.1 (1C, C-5a), 79.6 (1C, C-2a), 100.6 (1C, C-1a), 103.8 (1C, C-1b), 175.9 (1C), 179.4 (1C), 181.2 (1C), 185.5 (1C), 191.1 (1C) ppm

HRMS : calculated mass: 552.1931, found: 552.1933

Synthesis of nanoparticles

Silylation procedure of the porphyrin

(5-{p-[3-(2′,5′-dioxo-2′,5′-dihydro-1H-pyrrol-1′-yl)-N-3-phenoxypropyl)propanamide]-phenyl}-10,15,20-tri-p-pyridyl-porphyrine) precursor was silylated overnight at room

temperature following the procedure already described.[1] Briefly, 5.31 mg (6.31 μmol) of the porphyrin precursor were dissolved in 1 mL of MeOH and 4.3 μL (21.8 μmol) of (3-mercaptopropyl) trimethoxysilane were added. The reaction was stirred at room temperature for 12 h.

MSN

A mixture of CTAB (343 mg, 9.10^{-4} mol) and a freshly prepared solution of NaOH 0.12 M (20 mL) was stirred at 25°C for 2 hours at 400 rpm in a 500 mL three neck round bottom flask. The photosensitizer PS (1 mL, $6.31.10^{-3}$ mmol) was added and after 3 min, TEOS (1.75 mL, $0.78.10^{-2}$ mmol) was added dropwise. After 40 s, the mixture was diluted with 130 mL of deionized H_2O at 25°C and the stirring was increased to 750 rpm. The reaction was stirred for 6 minutes then rapidly neutralized to pH 7 by the addition of 0.2 M HCl (about 20 mL). Afterwards, MSN were obtained by centrifugation (20 min. 20000 turns/min.). CTAB was extracted by sonication with 30 mL of a solution of NH_4NO_3 in EtOH (6g/L) at 40°C. After centrifugation, the extraction procedure was repeated twice, and then MSN were put in suspension in EtOH and centrifugated three times. MSN were dried under vacuum for few hours and characterized by UV-visible spectra, with $\varepsilon = 62921\ M^{-1}.\ cm^{-1}$ for the Soret band ($\lambda = 415$ nm).

MSN-NH$_2$

MSN (75.6 mg) were put in suspension in H_2O (1.8 mL) for 20 min. EtOH (756 μL) and APTES (118 μl) were added. The suspension was neutralized to pH 6 by addition of HCl 0.2M. The reaction was stirred at RT for 20h, and MSN-NH_2 were centrifuged for 15 min at 20000 turns/min. The nanoparticles were washed by EtOH (dispersion with ultrasounds followed by centrifugation) and dried under vacuum for few hours.

MSN-M6C

MSN-NH$_2$ (9.4 mg) were put in suspension in EtOH (2.4 mL) for 10 min in sonication. **M6C** (1.3 mg, $3.3.10^{-3}$ mmol) was dissolved in H_2O (2 mL). This solution was added dropwise to the suspension of MSN-NH_2 in EtOH. The suspension was stirred at 50°C and 500 rpm overnight. After centrifugation (12 min, 10000 turns/min) the **MSN-M6C** were redispersed in water (ultrasounds) and centrifuged again (3 cycles). Then MSN-M6C were

redispersed in EtOH and centrifuged (two cycles). **MSN-M6C** were dried under vacuum for few hours and characterized by UV-visible spectra, with ε = 16000 M^{-1}. cm^{-1} for the ligand band (λ = 290 nm).

Amount of M6C grafted on the surface: 352 µmol.g^{-1}

MSN-M6C-Man

MSN-NH_2 (9.2 mg) were put in suspension in EtOH (2.4 mL) for 10 min in sonication. M6C-Man (1.83 mg, 3.3.10^{-3} mmol) were dissolved in H_2O (2 mL). This solution was added dropwise to the suspension of MSN-NH_2 in EtOH. The suspension was stirred at 50°C and 500 rpm overnight. After centrifugation (12 min, 10000 turns/min) **MSN-M6C-Man** were redispersed in water (ultrasounds) and centrifuged again (3 cycles). Then MSN-M6C-Man were redispersed in EtOH and centrifuged (two cycles). **MSN-M6C-Man** were dried under vacuum for few hours and were characterized by UV-visible spectra, with ε = 16000 M^{-1}. cm $^{-1}$ for the ligand band (λ = 290 nm).

Amount of M6C-Man grafted on the surface: 329 µmol.g^{-1}

Characterization of the nanoparticles

TEM analysis was performed on a JEOL 1200 EXII instrument. Dynamic light scattering analyses were performed using a Cordouan Technologies DL 135 Particle size analyzer instrument. N2 adsorption isotherms were measured using a TRISTAR 3000 gas adsorption analyzer instrument. The specific surface area was determined using the BET method. UV-vis absorption spectra were recorded on a Hewlett-Packard 8453 spectrophotometer and the correction factors used here are those supplied by the manufacturer.

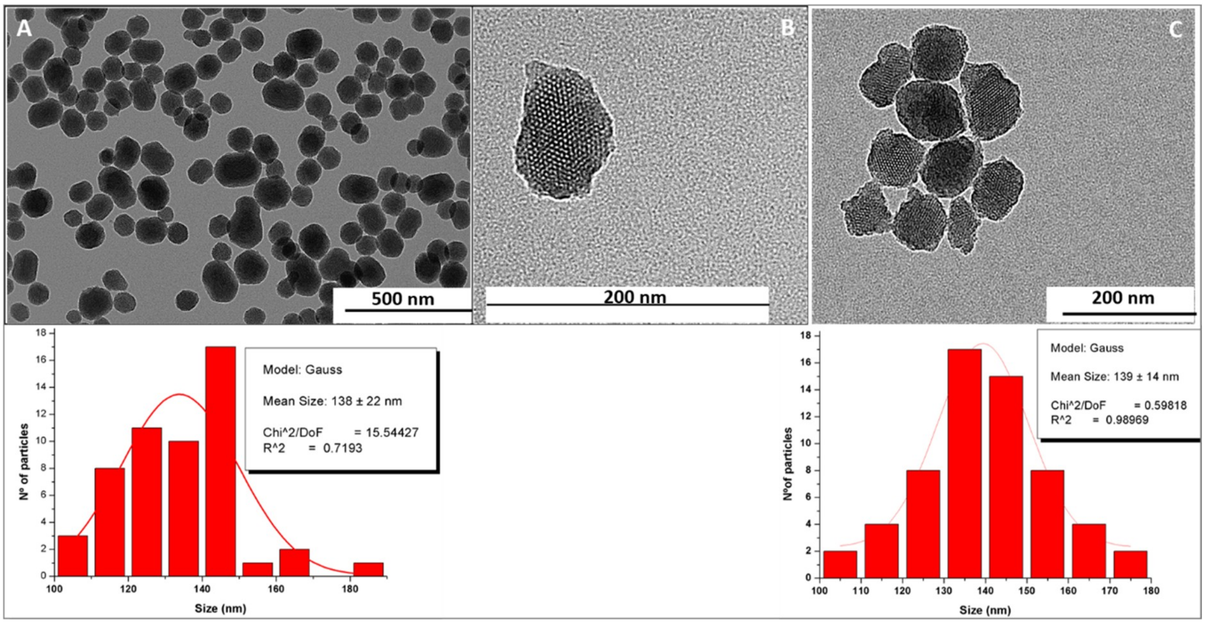

Figure S1. a) TEM image (scale bar: 500 nm) of MSN nanoparticles and their size distribution. 138 nm nanoparticles were obtained. b) The hexagonal structure of the porosity, characteristic of MCM-41 NPs. c) TEM image and size distribution of functionalized nanoparticles MSN-NH_2. (Scale bar: 200 nm).

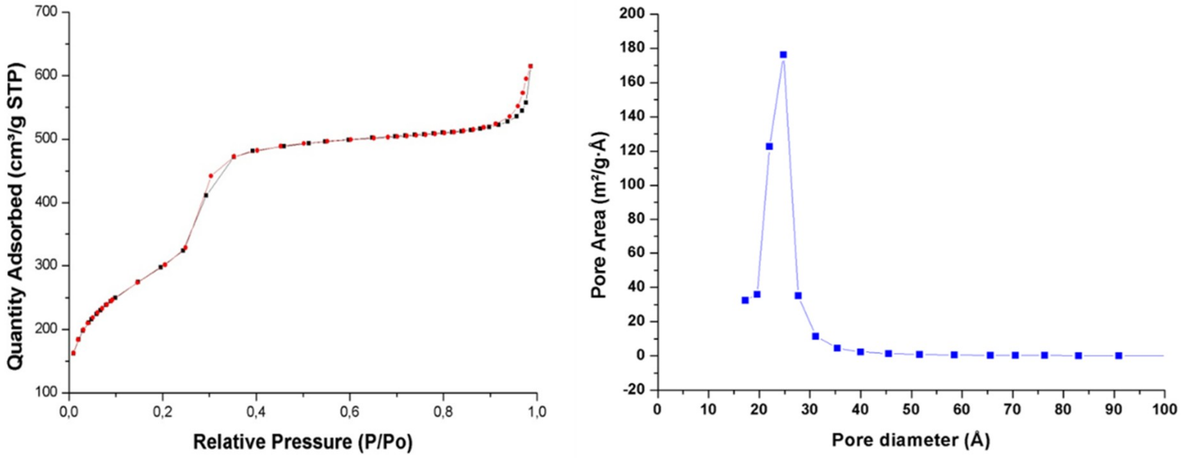

Figure S2. a) Nitrogen-adsorption desorption curve. MSN present a typical IV isotherm with a defined step at a relative pressure at 0.35. b) BJH analysis of pore diameter.

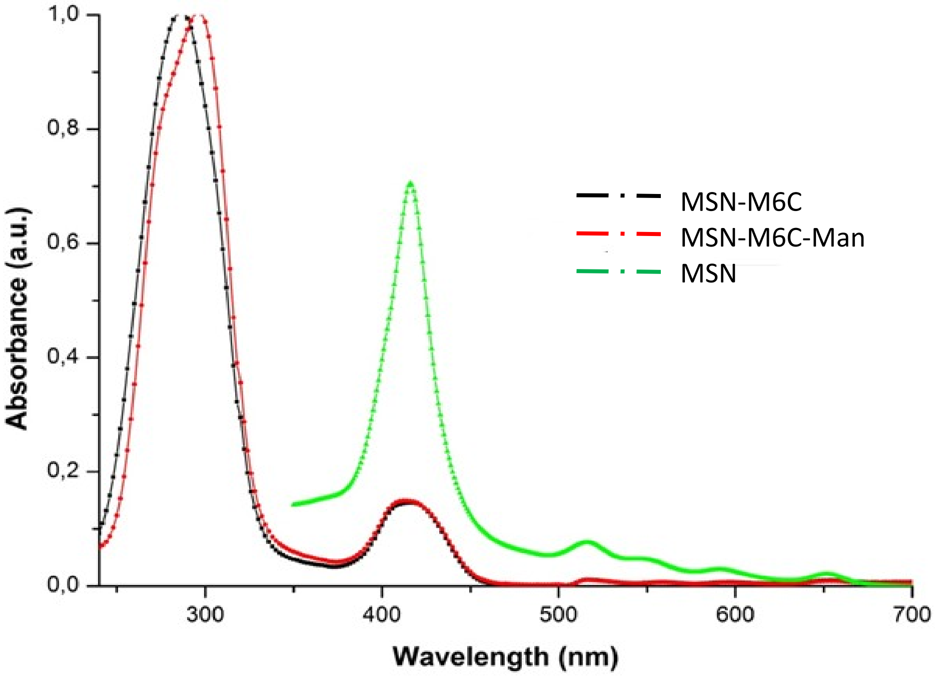

Fig. S3 UV-Visible absorption spectra of untargeted and targeted MSN.

1. Hocine, O.; Gary-Bobo, M.; Brevet, D.; Maynadier, M.; Fontanel, S.; Raehm, L.; Richeter, S.; Loock, B.; Couleaud, P.; Frochot, C.; Charnay, C.; Derrien, G.; Smaihi, M.; Sahmoune, A.; Morere, A.; Maillard, P.; Garcia, M.; Durand, J.-O., Silicalites and Mesoporous Silica Nanoparticles for photodynamic therapy. *Int. J. Pharm.* **2010,** 402, (1-2), 221-230. [doi: 10.1016/j.ijpharm.2010.10.004]
